# Underwater Robotics Competitions: The European Robotics League Emergency Robots Experience With FeelHippo AUV

**DOI:** 10.3389/frobt.2020.00003

**Published:** 2020-01-31

**Authors:** Matteo Franchi, Francesco Fanelli, Matteo Bianchi, Alessandro Ridolfi, Benedetto Allotta

**Affiliations:** ^1^Department of Industrial Engineering, University of Florence, Florence, Italy; ^2^Interuniversity Center of Integrated Systems for the Marine Environment, Genoa, Italy

**Keywords:** underwater robots, autonomous underwater vehicle, robotics competitions, autonomous navigation, acoustic mosaicing

## Abstract

Underwater robots are nowadays employed for many different applications; during the last decades, a wide variety of robotic vehicles have been developed by both companies and research institutes, different in shape, size, navigation system, and payload. While the market needs to constitute the real benchmark for commercial vehicles, novel approaches developed during research projects represent the standard for academia and research bodies. An interesting opportunity for the performance comparison of autonomous vehicles lies in robotics competitions, which serve as an useful testbed for state-of-the-art underwater technologies and a chance for the constructive evaluation of strengths and weaknesses of the participating platforms. In this framework, over the last few years, the Department of Industrial Engineering of the University of Florence participated in multiple robotics competitions, employing different vehicles. In particular, in September 2017 the team from the University of Florence took part in the European Robotics League Emergency Robots competition held in Piombino (Italy) using FeelHippo AUV, a compact and lightweight Autonomous Underwater Vehicle (AUV). Despite its size, FeelHippo AUV possesses a complete navigation system, able to offer good navigation accuracy, and diverse payload acquisition and analysis capabilities. This paper reports the main field results obtained by the team during the competition, with the aim of showing how it is possible to achieve satisfying performance (in terms of both navigation precision and payload data acquisition and processing) even with small-size vehicles such as FeelHippo AUV.

## 1. Introduction

Unmanned underwater vehicles, both teleoperated and autonomous, are nowadays employed for many applications, effectively helping human operators performing a wide variety of tasks (or even replacing them during their execution) (CADDY, Mišković et al., 2016). Underwater vehicles come in different shapes and sizes: from those with a length of several meters and a weight of hundreds of kilograms (e.g., Rigaud, 2007; Furlong et al., 2012; Kaiser et al., 2016) to the more compact and lightweight (for instance Hiller et al., 2012; Crowell, 2013; McCarter et al., 2014). While bigger vehicles naturally allow the use of more complex instrumentation and possess the ability to store heavy payload, smaller vehicles are commonly associated with lower performance and limited payload carrying capabilities. Hence, one of the current challenges that designers of small vehicles need to face consists in the optimization of the available space on board.

In this framework, the Mechatronics and Dynamic Modeling Laboratory (MDM Lab) of the Department of Industrial Engineering of the University of Florence (UNIFI DIEF) has been active in the field of underwater robotics since 2011, participating in different robotics-related research projects and developing and building several AUVs since then. Furthermore, throughout the years, UNIFI DIEF took part in multiple student and non-student robotics competitions. A team from UNIFI DIEF (UNIFI Team) took part in the Student Autonomous Underwater Vehicles Challenge - Europe (SAUC-E) Ferri et al. (2015) competition in 2012, 2013, and 2016, while in 2015 the team participated in euRathlon (Ferri et al., 2016); finally, it took part in the European Robotics Leaugue (ERL) Emergency Robots competition in September 2017 (Ferri et al., 2017).

This paper reports the field experience of the UNIFI Team at ERL Emergency Robots 2017, held in Piombino (Italy), from the 15th to the 23rd of September. During the nine competition days, the robots of the participating teams competed in a set of tasks in the land, air, and sea domains. This paper focuses on the results obtained in the sea domain with FeelHippo AUV: in particular, it will be shown how such vehicle, despite its small size, possesses a complete navigation system capable of offering satisfying accuracy while autonomously navigating; at the same time, it will be demonstrated how the diverse payload the vehicle is equipped with can be exploited for different purposes. In other words, the mechatronics design has been conceived to be a suitable trade-off between portability and high performance.

Other AUVs used in student robotics competitions can be found for example in Fietz et al. (2014) and Carreras et al. (2018) (the winner of ERL Emergency Robots 2017). The remainder of the paper is organized as follows: section 2 and section 3 are dedicated to the description of FeelHippo AUV; while the former focuses on the mechanical design of the vehicle and on the onboard devices, the latter describes its software architecture, giving an overview of its navigation system and describing some of its payload analysis and processing capabilities. Section 4 reports the most significant results obtained during the competition, and section 5 concludes the paper.

## 2. FeelHippo AUV: Description

FeelHippo AUV has been designed and developed specifically for the participation in student robotics competitions; it has been used by a team of UNIFI DIEF during SAUC-E 2013, euRathlon 2015, and ERL Emergency Robots in 2017.

In addition to student competitions, FeelHippo AUV has been used for short navigation missions, mainly in shallow waters, from 2015 onward. Thanks to the sensors added for the competition, the level of performance achieved was satisfying; hence, it was decided to incorporate such devices within the standard equipment of the vehicle. From early to mid 2017, FeelHippo AUV underwent a major overhaul, in terms of both mechanical components (identifying those parts and subsystems that could be redesigned to increase overall functionality) and navigation sensors (permanent integration of new instrumentation required indeed a general revision of the electronics of the vehicle, in order to optimize the occupied volume). In particular, the old oil-filled thrusters were replaced in favor of thrusters manufactured by BlueRobotics and tailored to underwater applications. In addition to this, a new DVL by Nortek has been placed under the center of gravity of the vehicle. Formerly, it was positioned in the stern. As a consequence, the stability of the vehicle is increased. More information concerning the payload can be found in the following. In its current version (as of end 2017, Figure 1), FeelHippo AUV can be efficiently used as a small survey and inspection AUV, suitable for use in present and future research projects or, generally speaking, autonomous sea operations.

**Figure 1:**
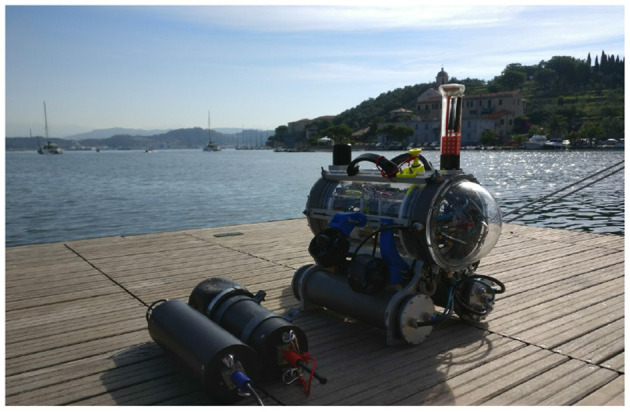
FeelHippo AUV, 2017 version.

The main characteristics of the vehicle are reported in Table 1; the reduced dimensions and weight, together with the convenient handles visible in Figure 1, allow for easy transportation and deployment (no more than two people are required, and even deployment from shore is possible).

**Table 1:** FeelHippo AUV physical data and performance.

**FeelHippo AUV main characteristics**	
Dimensions [mm]	~600 × 640 × 500
Mass [kg]	35
Max longitudinal speed [m/s] (kn)	~1 (2)
Max depth [m]	35
Autonomy [h]	2–3

The central body of FeelHippo AUV is composed of a Plexiglass^®^ hull with an internal diameter of 200 and 5 mm thickness, which houses all the non-watertight hardware and electronics. Two metal flanges constitute the connection between the central body and the Plexiglass^®^ domes at each end of the main hull, and two O-rings ensure a watertight connection between the former and the domes.

Four thrusters in vectored configuration (two on the stern and two on both lateral sides tilted of 45°), used to control translational motion and yaw (limited roll and pitch are guaranteed by hydrostatic stability), are connected with the central frame by 3-D printed custom-made plastic parts. Concerning the internal electronics, all the components are mounted on two parallel Plexiglass^®^ planes, placed on linear guides which facilitate assembly and maintenance operations (allowing to easily extract internal components from within the central body of the vehicle). An Intel i-7 Mobile CPU is used for onboard processing, while the sensor set FeelHippo AUV is equipped with includes:

- U-blox 7P precision Global Positioning System (GPS);
- Xsens MTi-300 AHRS, composed of triaxial accelerometers, gyroscopes and magnetometers;
- Nortek DVL1000 Doppler Velocity Log (DVL), measuring linear velocity and also acting as Depth Sensor (DS). The device has been placed under the central body of the AUV; indeed, being such component quite heavy (~2.7 kg in air), this choice increases stability in water;
- KVH DSP 1760 single-axis high precision Fiber Optic Gyroscope (FOG) for a precise measurement of the vehicle heading.

For what concerns communication, in addition to a WiFi access point and a radio modem, an EvoLogics S2CR 18/34 acoustic modem is used underwater; in addition, a custom-made antenna houses four rows of RGB LEDs, used for easy optical communication of the state of the vehicle (e.g., low battery, acquisition of the GPS fix, mission start) while the former is on surface. Regarding payload, the following devices are currently mounted on the vehicle:

- One Microsoft Lifecam Cinema forward-looking camera, which also allows teleoperated guide;
- One bottom-looking ELP 720p MINI IP camera;
- Two lateral ELP 1080p MINI IP cameras, used for stereo vision;
- One Teledyne BlueView M900 2D Forward-Looking SONAR (FLS).

**Figure 2:**
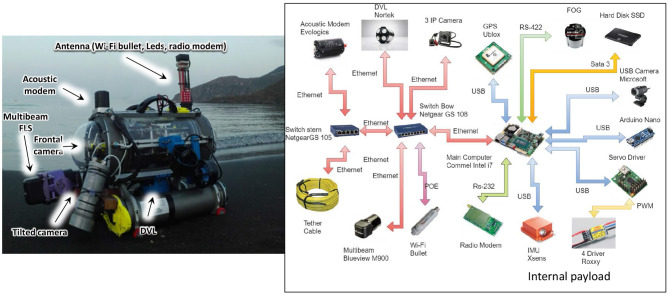
FeelHippo AUV connections scheme.

A scheme of the connections (logical and physical) among the components of the vehicle is reported in Figure 2. Despite its reduced size, FeelHippo AUV is able to equip diverse payload, both optical and acoustical. Furthermore, thanks to its particular structure, additional small devices (such as, e.g., supplementary cameras or LED illuminators) can be added to the main body of the vehicle with ease. More information about FeelHippo AUV versions from 2013 to 2017 can be found in Fanelli (2019), whereas more recent versions are described in Franchi et al. (2019). A comparison (in terms of dimensions and weight) with other competitors is reported in Table 2.

**Table 2:** FeelHippo AUV compared with other AUVs present on the market.

**AUV model**	**Dimensions (mm), weight (kg)**
FeelHippo AUV	600 × 640 × 500, 35
Remus 100 (Kongsberg)	1,700 × 190, 37
Sparus *II* (IQUA Robotics)	1,600 × 230, 52
LAUV (OceanScan-Marine Systems Technology)	150 × 2,300, 35

*The competitors are torpedo-shape vehicle and the dimensions are diameter × length*.

With the aim of highlighting the compactness of FeelHippo AUV, its physical data are compared with other AUVs present on the market.

## 3. FeelHippo AUV: Software Architecture

The software architecture is modular with independent processes that share information through an adapted TCP/IP protocol called Transmission Control Protocol for Robot Operating System (TCPROS) (Amaran et al., 2015; ROS). In section 3.1 a quick overview of the Guidance, Navigation, and Control (GNC) system is depicted, whereas in section 3.2 how to manage acoustic payload is described.

### 3.1. FeelHippo AUV: Guidance, Navigation, and Control System

Thanks to the available navigation sensors on board, introduced in section 2, FeelHippo AUV is capable of successfully performing autonomous navigation missions for the full extent of its battery charge without the need to resurface: thanks to a careful mechanical design, the vehicle is able to house position, depth, inertial, magnetic field, and velocity sensors inside its main body, thus disposing of a complete navigation system used to compute the pose of the AUV in real-time. Additionally, thanks to the presence of an acoustic modem, the vehicle is able to receive acoustic position fixes sent by dedicated instrumentation (e.g., Long, Short, or Ultra-Short BaseLine systems), which can be integrated within its GNC system and exploited to correct the pose estimated on board while underwater (or in any GPS-denied scenario).

The navigation filter of FeelHippo AUV is the same as the one of the others AUVs of the MDM Lab, exploiting all the features developed at the University of Florence during past and present research projects; hence, this section only briefly reviews the core concepts.

The navigation system is used to determine an accurate estimate of the pose of the vehicle with respect to a local Earth-fixed reference frame whose axes point, respectively, North, East, and Down (NED frame). Resorting to the classic notation exploited to describe the motion of underwater vehicles (Fossen et al., 1994), such quantity is denoted with $\eta ={\left[\eta_{1}\;\eta_{2}\right]}^{\prime }$, where **η**_1_ indicates the position of the AUV, and **η**_2_ its orientation (exploiting a triplet of Euler angles; roll, pitch, and yaw are used in this context). Additionally, let us denote with $\nu ={\left[\nu_{1}\;\nu_{2}\right]}^{\prime }$ the velocity (linear and angular) of the vehicle with respect to a body-fixed reference frame, and with **τ** ∈ ℝ^6^ the vector of forces and moments acting on the AUV.

**Figure 3:**
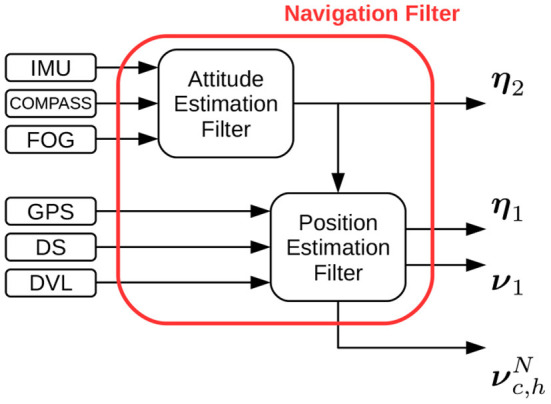
FeelHippo AUV navigation filter block scheme.

A parallel structure has been chosen (refer to Figure 3): attitude is independently estimated using IMU, compass, and FOG data, and constitutes an input that is fed to the position estimation filter. In particular, the attitude estimation filter is based on the nonlinear observer proposed in Mahony et al. (2008), whose principle is to integrate angular rate changes measured by gyroscopes and correcting the obtained values exploiting accelerometers and magnetometers. The structure of the original filter proposed in Mahony et al. (2008) has then been suitably modified in order to better adapt it to the underwater field of application (Allotta et al., 2015; Costanzi et al., 2016); in particular, a real-time strategy to detect external magnetic disturbances (which would detrimentally affect the yaw estimate) has been developed in order to maintain the accuracy of the computed estimate in a wide variety of possible environmental conditions, promptly discarding corrupted compass reading, and relying on the high precision single-axis FOG.

For what concerns position estimation, in addition to being able to navigate in dead reckoning (which has proven to be satisfyingly reliable despite its straightforward philosophy if the adopted sensors are sufficiently accurate), the vehicle can resort to an Unscented Kalman Filter (UKF)-based estimator. Such filter makes use of a mixed kinematic/dynamic vehicle model (so as to capture more information about the evolution of the system with respect to a purely kinematic model, but at the same time offering a reduced burden on the processing unit of the vehicle with respect to a complete dynamic model), taking into account longitudinal dynamics only (the majority of torpedo-shape AUV motion takes place on the direction of forward motion, since it usually constitutes the direction of minimal resistance).

The reader can refer to Allotta et al. (2016), Caiti et al. (2018), and Costanzi et al. (2018) for more details.

### 3.2. Payload Acquisition and Processing

Object detection and mapping is a typical problem in the underwater domain. Research on this topic is crucial for both AUVs and Remotely Operated Vehicles (ROVs), permitting them to understand their surroundings. Unfortunately, different and a priori unknown scenarios, which affect the robot-environment interaction, need to be faced. Poor visibility conditions in murky and turbid waters can compromise the operations of optical devices. To overcome the above-mentioned issues, FeelHippo AUV presents, as stated in section 2, a FLS. In the first part of the section, an acoustic-based buoy detection algorithm with a reinforcement that exploits the known geometric dimensions of a static target is proposed.

#### 3.2.1. FLS-Based Buoy Detection

**Figure 4:**
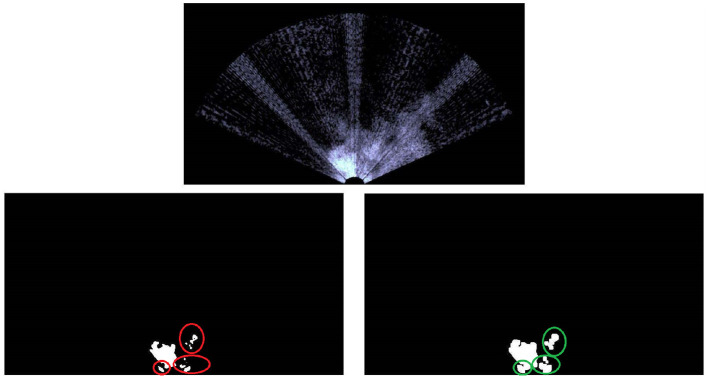
The image acquired by the FLS on top (note the bubbles in front of the vehicle that create a strong acoustic echo, see the white area). The binary threshold down on the left, whereas the latter is morphological dilated on the right. The red circles are the speckle areas and the green ones the subsequent aggregation.

The main concepts behind the algorithm are outlined:

- The acoustic video is acquired by one Teledyne BlueView M900 2D FLS and then it is real-time separated into a sequence of 8-bit grayscale images;
- Each frame is blurred with a Gaussian filter, leading to a smoother image.
- In order to detect high-reflection areas, which are likely to belong to a target object rather than to reverberation caused by the clutter, a direct binary threshold is applied to all the acoustic images. Let us define the source image as *src*, the destination image (namely the one after the binary threshold application) as *dst* and the threshold value *threshold* ∈ [0, 255]. Note that the interval limits depend on the depth of the image. As mentioned above, 8-bit grayscale images are considered;
- Each frame is modified by means of morphological dilations. Because of the environmental disturbances, some speckle areas, which do not belong to any buoy, can take place. Morphological operations make these areas to coalesce, so they can be easily ignored, avoiding false-positive detections. The situation is clearly visible in Figure 4 where high-reflection areas are due to the bubbles in front of the vehicle.
- At this point, several white colored bounded sets are present. Geometric boolean requirements need to be met to distinguish buoy-like objects from the background. The main assumption behind the proposed method lies in the knowledge (even rough) of the shape and dimension of the target to detect. On the one hand, our technique exploits simple geometric conditions; on the other hand, targets that resemble elementary geometric shapes are meant to be identified (circles, ellipses, rectangles). Commonly, typical buoys fall inside the scope of applicability of the proposed algorithm, which appears as a good trade-off. Four geometric properties that lead to four boolean conditions are considered and it is worth highlighting that all the requirements need to be met. First of all, the area of all the bounded sets is checked. If it is between a minimum (*a*_*min*_) and a maximum (*A*_*MAX*_) value, the condition is verified. The goal is trivial: ignore too small or too big regions. Second of all, the circularity, which is defined below, is investigated. If it is between a minimum and a maximum value, the condition is confirmed. Its meaning lies in understanding how much the bounded sets resemble a circle. Obviously, ellipticity is taken into account when circularity is different from one.
  (1) $$\begin{array}{ll} Circularity=\frac{4\pi A}{P^{2}}, & \; \end{array}$$
  where *A* is the area of the bounded set and *P* its perimeter. Afterwards, the convexity, defined as the ratio between the area of the set and the area of its convex hull (the smallest convex set that contains the original set), is checked. Another go/no go condition is applied.
  (2) $$\begin{array}{l} Convexity=\frac{A}{A_{ch}},\; \end{array}$$
  where *A* is the area of the bounded set and *A_ch_* is the area of the convex hull. It is easy to understand that convexity ∈ (0, 1].
  Lastly, the inertia ratio, which is defined in Equation (3) is verified. The goal is trivial: detect whether the object is elongated along a particular direction. Note that the moments of inertia are calculated with respect to the center of mass of the set.

(3) $$\begin{array}{l} IR=\frac{Imax}{Imin},\; \end{array}$$

where *Imax* and *Imin* are respectively the maximum and the minimum moment of inertia (the inertia along the principal axes) and *IR* ∈ ℝ^+^.

Unfortunately, as stated by Hurtós et al. (2015), FLS imagery are affected by low Signal-to-Noise Ratio (SNR), poor resolution and intensity modifications that depends upon viewpoint variations, so some false positive detections might arise anyway.

Assuming a static target (very often a buoy falls inside this class), a position-based clustering algorithm with the aim of removing false positives, can be exploited. If several detections are accumulated around a small region, then what is insonified by the FLS has high probability to be the buoy. In other words, the presence of spurious noise and mobile objects (e.g., fish) can be managed by the proposed technique, leading to a robust solution. The key idea exploits the solution proposed in Ester et al. (1996) where, basically, elements with many nearby neighbors are grouped together, whereas points that lie too far from their closest neighbors are classified as outliers.

To locate the exact position of the detected targets, starting from the known position of the vehicle, an imaging geometry model needs to be defined and the reader can refer to Franchi et al. (2018) for more information. In few words, exploiting the work of Johannsson et al. (2010), Ferreira et al. (2014), Hurtós et al. (2015), and Walter (2008), a simplified linear model, where the FLS can be treated as an orthographic camera, is adopted.

## 4. ERL Emergency Robots 2017 Experimental Results

**Figure 5:**
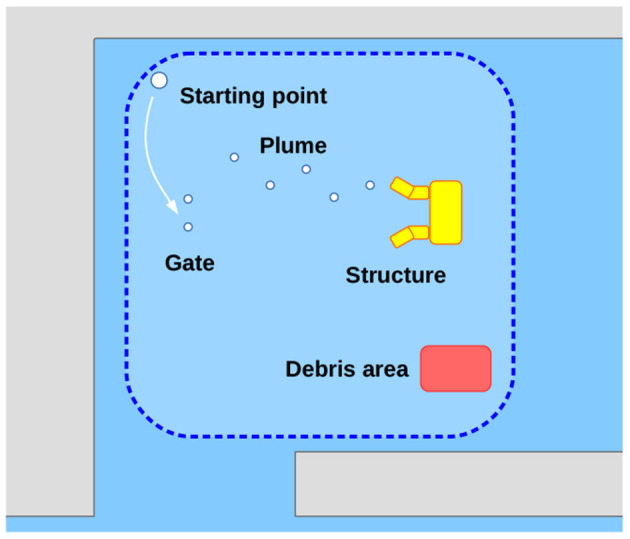
ERL Emergency Robots 2017 sea domain arena.

This section reports some of the results obtained during the robotics competition ERL Emergency Robots 2017, held in Piombino (Italy) in September 2017. In particular, the data shown here refer to multiple autonomous missions performed by FeelHippo AUV during the sea domain trials throughout the competition [refer to Ferri et al. (2017) for more details about the challenge]. Robots were asked to act in the following (recreated) catastrophic scenario: after an earthquake and a tsunami hit the shoreline area where a nuclear plant is located, evacuation procedures are issued; however, several people working at the plant are missing. Additionally, the premises have suffered damages of relevant intensity, with their lower sections flooded; furthermore, several pipes of the plant (both on land an underwater) are leaking radioactive material. Concerning the sea domain, the area of interest was constituted by a rectangular arena ~50 × 50 m wide. Beyond a starting gate, composed of two submerged buoys, lied the area of interest where an underwater plastic pipe assembly represented the (flooded) lower section of the plant. Obviously, no substance was actually leaking; a set of five numbered underwater buoys was used to represent the leaking fluid plume (leading to a particular component of the pipe assembly, where a specific marker represented the breakage). In addition, several objects anchored on the seabed (e.g., tables and chairs) indicated a debris area where it was likely to find the body (i.e., a mannequin dressed in easy visible orange) of one of the missing workers. See Figure 5 for a graphical representation of the arena and of the objects of interest (note that the picture is not to scale, and the positions of the depicted objects are not meant to represent actual shapes or dimensions).

Each participating team was allotted an exclusive time slot in the arena; from the starting point, the vehicle had to submerge, pass through the gate (without touching it, and providing optical or acoustical images of the gate itself), and it was then required to perform different tasks without resurfacing. Among the different tasks (but not limited to those mentioned here), each AUV was asked to inspect and map the area and the objects of interest (e.g., the plume, the gate, the underwater pipe assembly, the debris area) and to identify in real-time the mission targets, such as the leaking pipe and the missing worker. A specific score based on the degree of completeness and on the quality of the provided data (navigation and/or payload data, used to guarantee the veracity of team's claims on each submission) was assigned to each task. Hence, each AUV had to (a) precisely navigate through the arena, closely following the planned path in order to (b) efficiently make use of its own payload and payload processing algorithms, mapping the arena and identifying the objects of interest during navigation so as to score as much points as possible. In light of the above-mentioned considerations, this section is divided into two parts: at first, the focus will be given to the navigation performance of the vehicle, showing how FeelHippo AUV is able to follow a desired trajectory without incurring in an unacceptable position estimation error growth over time; then, it will be shown how the payload the vehicle is equipped with can be suitably used to accomplish the goals of the competition. Despite of the reduced size, its optimized mechatronics design, indeed leads to a compact but high-functional vehicle.

### 4.1. Navigation Results

The results reported in this section refer to the mission performed by FeelHippo AUV during the final trial of the competition; hence, the path executed by the vehicle was planned according to the estimated positions of the objects of interest, evaluated from the in-water runs executed during the previous days. In particular, after passing through the gate, the vehicle autonomously performed a lawnmower path with West-East aligned transects, to cover as much as possible of the area of interest. Then, a second lawnmower path, perpendicular to the first, was executed in the northern part of the arena, where the plume buoys were supposed to be. Two final waypoints were included in the direction of the debris area in order to try to identify the objects composing the area itself or even the mannequin representing the worker.

**Figure 6:**
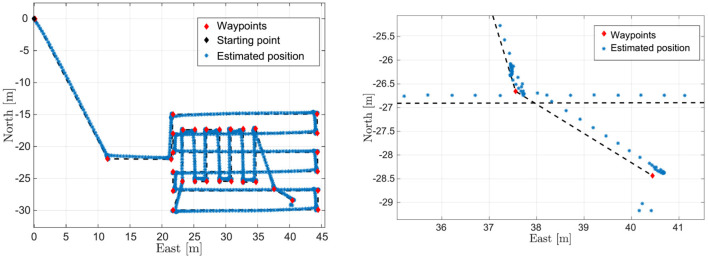
On the left, FeelHippo AUV estimated path, whereas on the right focus on the resurfacing position.

Figure 6 shows the position estimate computed by FeelHippo AUV during the execution of the autonomous mission. The first waypoint (the starting point of Figure 5) was located at 42.954164° N, 10.6018952° E; the task was executed at the desired depth of 1 m (except for the last two waypoints, located at the depth of 3.5 m), with a desired longitudinal speed of 0.5 m/s and a covered path of about 240 m. The discontinuity visible in the lower-right corner of Figure 6 is due to the error between the path estimated onboard the vehicle while navigating underwater and the GPS fix acquired after resurfacing. Indeed, such error is <1 m after about 21 min of navigation (or, equivalently, <1% of the total length of the path), highlighting the satisfying accuracy of the navigation system of the vehicle: it is worth remembering that FeelHippo AUV performed the whole underwater mission autonomously, without resurfacing; communication from the ground control station to the vehicle (exception made for mission starts and possible emergency aborts) was specifically forbidden by the competition rules.

### 4.2. Payload Processing Results

FeelHippo AUV was asked to autonomously (and possibly real-time) find the seven buoys located in the sea domain arena, see Figure 5. Their physical characteristics in terms of color (orange), shape (approximately spherical) and dimensions (radius around 0.3 m) were a priori known.

**Figure 7:**
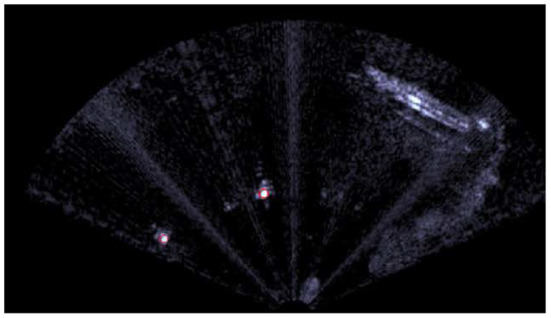
The starting gate, the red circles on the target buoys state the detection.

**Figure 8:**
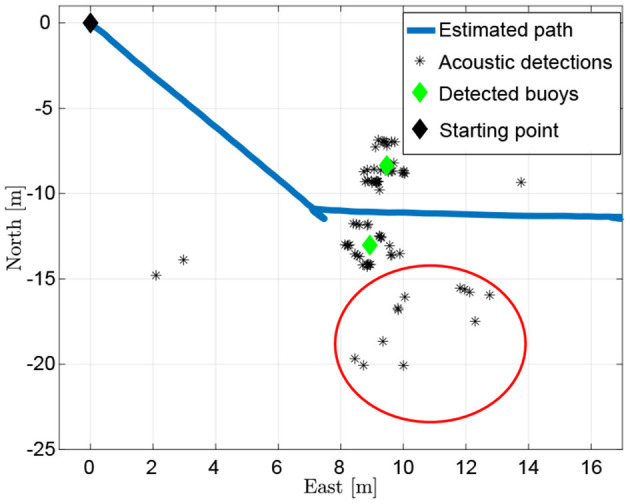
The acoustic detection of the starting gate with the aid of the clustering algorithm.

While FeelHippo AUV was performing the path described in section 4.1, the buoys detection took place. The starting gate, composed of two buoys, is visible in Figure 7, whereas the result of the proposed solution is depicted in Figure 8. In the former, the rubber boat where the judges monitored the course of the competition can be noticed on the top-right corner. In the latter, due to the presence of the rubber boat, false positive detections take place (note the red circle). On the other hand, given their scattered nature, the clustering algorithm is able to handle the situation. In particular, before applying the clustering algorithm 82 detections take place, where 67 are true positives and 15 false positives. It is worth highlighting that the detection operation, as well as the target geolocalization, were conducted in real-time and any geometric constraints has been exploited for target detection (for example, the known geometric distance between the buoys that compose the starting gate).

**Figure 9:**
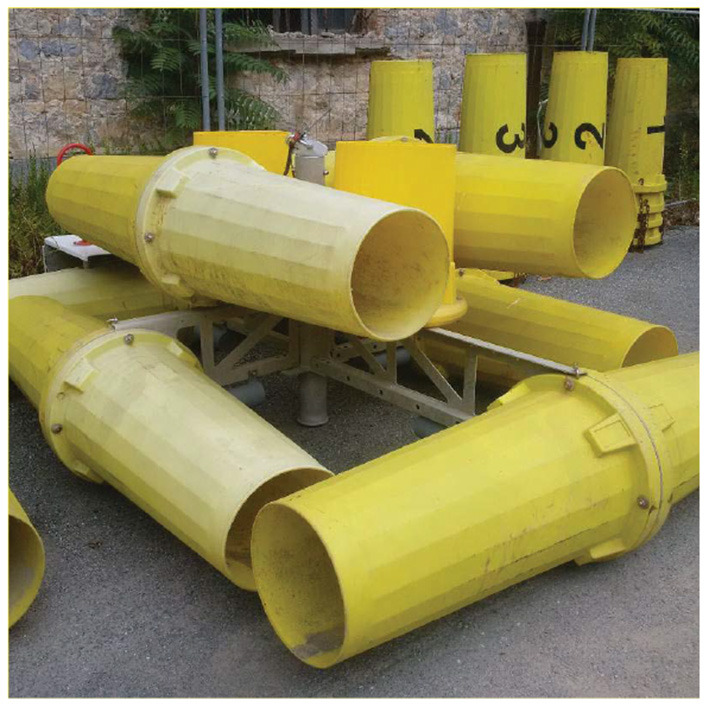
The structure placed on the sea bottom (Ferri et al., 2017).

**Figure 10:**
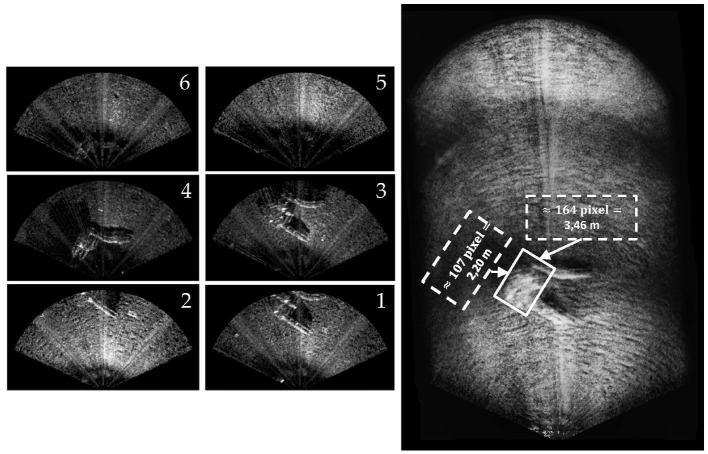
The 2D mosaic of the underwater structure. On the right, the dimension of the underwater structure (retrieved by means of the mosaic) is reported.

After the end of the competition, a 2D mosaic of the area around the structure (see Figures 5, 9), namely an underwater plastic pipe assembly with the aim of representing the (flooded) lower section of the plant, was performed. For a detailed description of the acoustic mosaic formation process, the interested reader can refer to Franchi et al. (2018). The proposed solutions make use of the OpenCV library (OpenCV). To this end, a new mission, where the FLS was mounted with a small tilt angle (~20° with respect to the water surface), was executed. The collected dataset was composed of 72 FLS images recorded along a 20-meter transect. The maximum FLS range was set to 10 meters and the FOV of the device was 130° (uneditable by the user). A few FLS frames and the final composite are reported in Figure 10. In the latter, the covered area is ~500 m^2^. Furthermore, the real dimensions of the underwater structure (which were a priori known) are in accordance with the size that can be obtained from Figure 10. Indeed, structure dimensions are about 2.20 × 3.20 × 1.20 m, whereas the obtained ones are 2.20 × 3.46 m. More information concerning the conversion from pixels to meters is presented by the authors in Franchi et al. (2018).

## 5. Conclusion

The paper shows how FeelHippo AUV, despite its small size, represents a compact and complete underwater platform, which can be employed in different application scenarios.

In particular, a reliable and versatile navigation system, able to perform satisfying accuracies, is shown in section 3.1; indeed, two navigation approaches (the vehicle can exploit a dead reckoning strategy as well as a UKF-based solution) that present a relative error <1% after about 21 min of autonomous navigation are proposed.

For what concerns the payload acquisition and processing, an acoustic-based object detection algorithm (in our case, applied to underwater buoys) is treated in section 3.2.1, where substantial improvements through clustering techniques (usable in presence of static targets) are presented (Figure 8). Good performance in terms of detection even with limited visibility ranges are shown and, in addition, the real-time implementation is proposed. Lastly, an underwater acoustic mosaic is presented in section 4.2. The presented solution is shown to perform satisfying 2D underwater reconstruction of the order of hundreds of square meters. Future works will involve machine learning-based detection techniques and a mixed detection approach that resorts to a FLS and an optical camera.

The UNIFI Team has been awarded Second-in-Class in “Pipe inspection and search for search for missing workers (Sea+Air)” during ERL Emergency Robots 2017.

## Data Availability Statement

The datasets generated for this study are available on request to the corresponding author.

## Author Contributions

MF: algorithm development, experiments, results validation, and writing. FF: experiments and results validation. MB: experiments and writing. AR: results validation, writing, and activities supervision. BA: writing and activities supervision.

### Conflict of Interest

The authors declare that the research was conducted in the absence of any commercial or financial relationships that could be construed as a potential conflict of interest.
